# Stress and Stressors Among PHCC Dentists: A Quantitative, Correlational, and Cross-Sectional Study

**DOI:** 10.3390/ijerph21121581

**Published:** 2024-11-27

**Authors:** Kholoud Z. Abbas, Najat A. Alyafei, Arwa S. Tayyem, Mohammad R. Zakarya, Hamad R. Al Mudahka, Abdel-Salam G. Abdel-Salam, Hashim A. Mohammed

**Affiliations:** 1 Dentistry Department, Primary Health Care Corporation, Doha P.O. Box 26555, Qatar; 2 Preventive Health Directorate, Primary Health Care Corporation, Doha P.O. Box 26555, Qatar; 3 Oral Surgery, Primary Health Care Corporation, Doha P.O. Box 26555, Qatar; mzakarya@phcc.gov.qa; 4 Department of Mathematics, Statistics and Physics, CAS, Qatar University, Doha P.O. Box 2713, Qatar; abdo@qu.edu.qa; 5 Family Medicine, Primary Health Care Corporation, Doha P.O. Box 26555, Qatar; hasmohamed@phcc.gov.qa; 6 Weill Cornell Medicine-Qatar, Doha P.O. Box 24144, Qatar

**Keywords:** perceived stress, job stress, dentists, Primary Health Care Corporation in Qatar (PHCC)

## Abstract

(1) Background: Dentistry is innately stressful and demanding. However, the extent of perceived chronic stress and the contributing factors within the dental practice at Primary Health Care Corporation (PHCC) in Qatar are largely unknown. (2) Methods: This study is quantitative, hypothesis-testing, correlational, and cross-sectional, using a simple random sample of all PHCC dentists (168 general dentists and 47 specialist dentists). A cross-sectional survey with electronic consent was performed using demographic and professional information, the Perceived Stress Scale (PSS)-10 to evaluate chronic stress, and the Job Stress Inventory (JSI) to assess job stress factors. (3) Results: The response rate was 49.3%. PHCC dentists demonstrated above-average perceived stress (mean = 18.08, SD = 5.84), with a significant relationship with age (*p* = 0.01). Overall job stress was average (mean = 2.45, SD = 1.18). All job factors demonstrated a significant and positive correlation with perceived stress (r = 0.472–0.555, *p* < 0.001). (4) Conclusion: About 70% of the dentists experienced high levels of chronic stress that decreased with age; 65.4% of this stress was attributed to job-related factors, primarily those involving patients (β = 0.341, *p* < 0.001). This study highlights the impact of health system reforms on stress levels among dental professionals in primary care settings.

## 1. Introduction

Stress is a universal phenomenon that affects the general health of many people due to numerous personal, communal, and ecological interactions with the surrounding environment (Shahsavarani et al., 2015) [1]. The influence of depressive disorders extends to several professional domains (Dehnad et al., 2016) [2], especially among individuals with certain personality traits, such as dentists (Rada and Johnson 2004) [3]. The term “stress” has a far-reaching range of definitions, and several theories have been put forward to explain its dynamics; Hans Selye (1956) presented stress as a “response” to variable stimuli that accentuate three phases, including “alarm” and “resistance” to stress to regain “homeostasis”, recognized as the general adaptation syndrome (Robinson 2018) [4]. The “stimulus” approach by Holmes and Rahe (1967) [5] considers grueling events like marriage or death to be a threatening “stimulus” that challenges the organism and instigates cumulative and variable adverse influences. The transactional stress theory by Lazarus and Folkman (1984) [6] associates stress with the interaction between an individual and the environment through cognition, appraisal of the threat, and effective coping—while insufficient stress coping maneuvers, certain personality traits, and limited resources to mitigate the “demand” can lead to the emergence of stress (Smith and Lazarus 1990) [7].

Perceived stress refers to feelings of a lack of control, the unpredictability of the future, and the frequency of troublesome problems (Whittaker Was Phillips 2015) [8]. This type of stress can be evaluated by the frequency of these encounters (Cohen et al., 1983) [9], indicating a chronic psychological state rather than a temporary condition (Mathur et al., 2016) [10], while acute stress pertains to everyday stress that may intensify in response to traumatic events (Siedlecki 2013) [11].

Work-related stress, on the other hand, is a reaction to job demands and pressures that exceed people’s knowledge and abilities, thereby challenging their coping capacity in text (ILO 2022) [12]. Whether or not dentistry is more stressful than other health professions, dentistry is a demanding occupation that requires physical and mental effort to maintain consistent and high-quality dental care for recursively anxious patients (Pouradeli et al., 2016) [13]. These severe work conditions could potentially have unfavorable consequences on personal and professional lives (Queirolo et al., 2023) [14], exposing dentists to an increased risk of occupational stress and burnout (Goetz et al., 2012) [15]. Dentists also report difficulties in managing stress and suicidal thoughts (Collin et al., 2019) [16].

Findings obtained from research indicate that stress factors associated with dental practice are unique and distinctive; this is also evident when stress-causing factors in private and public care are explored (Wood 2021) [17]. Uncooperative patients and concern about unsuccessful treatment results can be challenging for specialist dentists (Kim et al., 2020) [18]. At the same time, general dentists may feel pressured to remain focused and strive to manage their time effectively (Pouradeli et al., 2016) [13].

Research conducted in Arab countries consistently highlights stress as a significant health issue. In Syria, approximately 62% of dentists who participated in a survey indicated feeling stressed due to the challenge of sustaining persistent concentration (Almasri 2020) [19]. In Yemen, dentists experience stress primarily due to uncooperative patients, workload, and the pursuit of technical perfection (Al-Zubair et al., 2015) [20]. On the other hand, Lebanese dentists mostly feel stressed when patients arrive late for their appointments (DAOU et al., 2022) [21].

Stress factors are evolving, reflecting how practice settings change over time (Collin et al., 2019) [16]. These changes uncover the global impact of stress on dental professionals and emphasize the need to recognize the stress experienced in various dental practices. This recognition is essential for effectively supporting and overseeing the emotional well-being of dental practitioners. However, the prevalence of perceived stress among dental professionals and the effect of working in a dental environment on the chronic stress levels of dentists at PHCC have not been thoroughly investigated.

In this study, the following null and the alternative hypotheses will be used to evaluate the work-related stress of PHCC dentists.

Ho: Chronic stress perceived by dentists is unlikely to be correlated with stress at work.

Ha: Chronic stress perceived by dentists is likely to be correlated with stress at work.

This research aims to assess the correlation between perceived chronic stress and work-related stress in a cohort of dentists working in Primary Health Care Corporation (PHCC).

## 2. Materials and Methods

### 2.1. Research Design

An anonymous cross-sectional survey using simple random sampling was performed. All questions were marked mandatory. Participants consented by clicking the “I Agree” button. They could also withdraw from the survey. The questionnaire was exclusively accessed through official email to ensure that only PHCC dentists could participate.

Inclusion criteria: all those who are PHCC dentists.

Exclusion criteria: all those who are not PHCC dentists.

### 2.2. Sample

The sample size for this study was estimated using a confidence level of 95% with a 5% margin of error and a population of 40% or more. A total of 137 or more dentists were required for the study. The dentists were recruited by email after the necessary approvals were obtained.

### 2.3. Measurements

The online survey consisted of three parts, as described below.

#### 2.3.1. Sociodemographic Variables (Lee et al., 2014) [22]

The list of sociodemographic variables was adapted from the Korean version of “Perceived Stress Scale (PSS-14, 10 and 4): psychometric evaluation in patients with chronic disease”. Each PHCC dentist’s specialty, role (dental lead or not), gender, age, marital status, time of work at PHCC, engagement in physical activity, hours of sleep, and medical status were examined.

#### 2.3.2. Perceived Stress Scale (Cohen et al., 1983) [9]

The 10-item PSS measures how much certain incidents impacted perceived stress during the previous month on a 5-point Likert scale: never (0), almost never (1), sometimes (2), fairly often (3), very often (4). The PSS minimum value is 0, and the highest value is 40. Scores were obtained by reversing the responses of the positively expressed statements (items 4, 5, 7, and 8) (e.g., 0 = 4, 1 = 3, 2 = 2, 3 = 1, and 4 = 0) and then summing the coded Likert scale (1, 2, 3, 4R, 5R, 6. 7R, 8R, 9, 10). The higher scores indicate greater psychological stress. The average scores on the PSS were interpreted so that 0–7 = very low; 8–11 = low; 12–15 = average; 16–20 = high; and 21 and over = very high (Bhat and Nyathi 2019) [23]. The accepted scale reliability is set at a Cronbach’s alpha above 0.70.

#### 2.3.3. Job Stress Inventory (JSI) (Hosanguan 2007) [24]

The JSI is a thirty-item tool that describes job stressors categorized into five constructs or factors. The JSI was reviewed by the researchers and by well-experienced dentists, who agreed to replace item 8, item 19, and item 22 with “managing children”, “being supervised by several managements”, and “causing pain to patients”, respectively. The JSI questions ask how one feels about each item listed on a 5-point Likert scale (1 = least stress, 2 = little stress, 3 = moderate stress, 4 = very stressful, 5 = most stress). The JSI mean scores range between a minimum of 1 and a maximum of 5, and higher scores indicate higher levels of job stress. The average scores on the JSI were organized into four categories: low (1–1.99), average (2–2.99), high (3–3.99), and very high (4–4.99). The internal consistency for group comparisons was considered satisfied if a Cronbach’s alpha coefficient ≥ 0.70 was achieved according to the primary validation of the JSI.

#### 2.3.4. Variables in the Study

Job stress levels were considered the dependent variable, while perceived stress levels were the independent variable. The severity of perceived stress was expected to vary according to different stress factors. Sociodemographic variables were included as confounding factors in the study.

### 2.4. Statistical Analysis

The results were collected into Excel sheets and analyzed by the Statistical Package for Social Sciences (SPSS) version 26.0. Descriptive statistics, hypothesis testing, and regression analysis were conducted.

#### 2.4.1. Perceived Stress Scale (PSS-10 Items)

i. Descriptive statistics were utilized to describe the basic features of the data; frequencies and percentages provide insights into the distribution of categorical responses. Means and standard deviations were used to detect variability.
ii. The Chi-square test was utilized to detect associations between two categorical variables (e.g., male vs. female) and a categorical representation of stress levels (high vs. low), based on the respondents’ scores. The Chi-Square test is useful when dealing with categorical variables and when the expected relationship is linear.
iii. One-way ANOVA (F-statistics) was applied to make comparisons across three or more groups (e.g., age groups, job roles). The significance of the findings was determined using a *p*-value < 0.05.

#### 2.4.2. Job Stress İnventory (JSI)

i. Descriptive statistics: means and standard deviations were used to detect variability.
ii. Confirmatory factor analysis (CFA) was used to validate the factor structure of the JSI and assess to what extent each factor was predictive of the work-related stress of PHCC dentists, while internal consistency was detected by Cronbach’s alpha at a threshold of ≥0.70, with the significance of findings determined using a *p*-value < 0.05, indicating that each item within the JSI reliably measures the underlying factor.
iii. Regression analysis was used to assess the linear relationship between scaled variables (e.g., JSI scores vs. age, JSI scores vs. PSS scores), with significance set at *p*-value < 0.05.

## 3. Results

Table 1 presents the demographic information of the participants. Out of the total population who received the emails, 44.6% of general dentists and 66.0% of specialist dentists completed the survey. Initially, 107 responses were collected. However, one participant opted to stop the survey, resulting in a total of 106 respondents who successfully answered all the questions. The response rate stood at approximately 49.3%; the participation rate of each specialty exceeded 30%. The survey findings indicated that 70.8% of the respondents were general practitioners, 14.2% specialized in pediatric dentistry, 20.8% held leadership positions in dental care, and 57.5% were male. The majority of participants were married, accounting for 93.4% of participants, and were in the age range of 30 to 45 years, comprising 62.3% of the group. Participants aged between 46 and 60 closely followed, making up 37.7%, whereas individuals above 60 years old constituted a smaller fraction of the total population at 2.8%. Additionally, 34.0% of individuals had accumulated up to 5 years of professional experience at PHCC. Most of the participants (71.7%) engaged in some form of physical activity. In terms of health conditions, the majority (67.9%) did not report any medical issues, whereas 12.3% of the dentists experiencing health problems reported hypertension or cardiovascular disease. In terms of sleep duration, it was found that the vast majority of participants (95.3%) slept for less than 8 h each day, with a small percentage (4.7%) reporting more than 8 h of sleep.

As shown in Table 2, upon conducting a descriptive analysis, it was found that total PSS score for the 106 participants had a mean = 18.08, SD = 5.87, and a median of 18, while the minimum value was 4 and the maximum was 38. Overall, the mean perceived stress values showed variation among different categorical groups, with the lowest value observed for dentists sleeping over 8 h (mean =15.60, SD = 4.83) and the highest for new employees with up to 5 years of experience (mean = 19.28, SD = 6.23). The average perceived stress levels showed a decline as age increased. Dentists aged between 30 and 45 exhibited higher stress levels, with a mean = 19.21 and SD = 5.47, compared to dentists over 45 years old, who had a mean = 16.23 and SD = 6.11. In terms of PSS score, dentists over 60 years of age had a mean = 12.33 and SD = 6.03. The correlation coefficient indicates that sociodemographic factors do not have a considerable influence on the severity of perceived chronic stress levels (*p* > 0. 05), except for age group (*p* < 0.05).

As shown in Table 3, an ANOVA was performed to determine whether there are significant variations in PSS scores among the three age groups. The F-statistic of 6.80163 and the *p*-value < 0.05 indicate that variations in age group have a significant influence on stress levels.

Eta squared (η^2^) for ANOVA and effect sizes for a *t*-Test were calculated for age groups. η^2^ = 0.0614, meaning that approximately 6.14% of the total variance in PSS scores is explained by age group differences.

The calculated Cohen’s d is approximately 0.69. This value indicates a moderate effect size for the difference in perceived stress scores between the age groups, suggesting that the age group difference has noticeable, though not large, practical significance.

**Figure 1:**
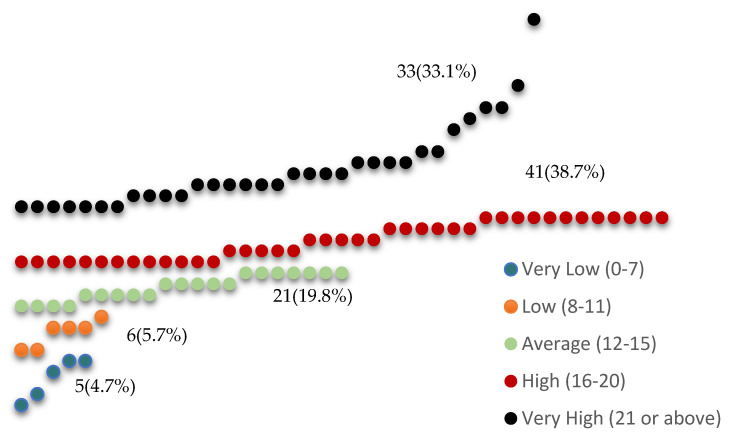
Distribution of PHCC dentists according to perceived stress levels.

Figure 1 represents the distribution of the dentists according to the severity of perceived stress levels organized into five categories: very low, low, average, high, and very high. The mean PSS scores for each respective category show varying results: very low—5.40 (SD = 1.82); low—9.50 (SD = 1.22); average—13.71 (SD = 1.15); high—17.98 (SD = 1.67); and very high—24.48 (SD = 3. 80). The combined high and very-high PSS scores indicate that approximately 70% of PHCC dentists have an above-average perceived stress level.

On the whole, PHCC dentists showed moderate levels of job stress, with an average of 2.66, a standard deviation of 1.15, a minimum of 1.00, and a maximum of 4.33. Cronbach’s alpha values revealed a high level of consistency across the entire scale at 0.95, with subscale reliability ranging from 0.73 to 0.91.

From Table 4, it can be seen that the primary stressors showed considerable significance various job stress factors were tested, resulting in five key categories: patient-related (9 items), significant at *p* < 0.05; job conditions (5 items), significant at *p* < 0.05; health system reforms (6 items), significant at *p* < 0.05; and job characteristics (4 items), with a level of *p* < 0.05. On the other hand, statistical nonsignificance was observed concerning time pressure (6 items), with *p* > 0.05. Patient-related factors accounted for 43.42% of dentists’ job stress, while the overall JSI accounted for 65.39% of stress observed among PHCC dentists.

One-way ANOVA was utilized to evaluate the statistical significance of the variables under investigation, revealing a *p*-value < 0.05. The primary stressors within the PHCC dental environment showed a high level of significance when the pertinent job stress factors were examined: for patient-related stressors, F(8, 945) = 3.15, *p* < 0.05; for job conditions, F(4, 525) = 2.48, *p* < 0.05; for health system reforms, F(5, 630) = 4.07, *p* < 0.05; and for job characteristics, F(3, 420) = 4.37, *p* < 0.05, indicating statistically significant effects on the study participants. The analysis indicates that time constraints were statistically insignificant, as indicated by F(5, 630) = 0.75, *p* > 0.05.

As shown in Table 5, the Pearson correlation coefficient demonstrates the relationship between job stress and perceived stress across various factors. For Factor 3 (health system reforms—6 items), the correlation was r(104) = 0.555, *p* < 0.001. Factor 1 (patient-related—9 items) showed a correlation of r(104) = 0.533. Factor 2 (job conditions—5 items) had a correlation of r(104) = 0.472, *p* < 0.001. Factor 5 (time pressure—6 items) displayed a correlation of r(104) = 0.499, *p* < 0.0001, while Factor 4 (job characteristics—4 items) had a correlation of r(104) = 0.476, *p* < 0.001. These results indicate a significant positive association between general stress and job stress, with a *p*-value of <0.001. Furthermore, the findings suggest that as stress intensifies due to health system and patient-related issues, job stress levels increase by 55.5% and 53.3%, respectively. The observed relationship between job stress factors and the Perceived Stress Scale (PSS) based on the hypotheses is consistent and not random, with r = 0.607, *p* < 0.001.

Table 6 represents a regression analysis of the JSI conducted against the five JSI factors; the results show that four of them (patient-related, job condition, health system reform, and time pressure) are highly significant (R^2^ ≈ 0.99). The results indicate that the four factors account for approximately 99.9% of job stress.

Table 7 presents the ANOVA results for a regression model with Job Stress Index (JSI) as the dependent variable, analyzed using a linear regression through the origin (i.e., without a constant term). The model includes four predictor variables labeled F1, F2, F3, and F5. The resulting F-statistic of 6.80163, with a *p*-value of 0.000, suggests that the overall model is statistically significant at the conventional levels, meaning that at least one of the predictors contributes meaningfully to explaining the variation in JSI.

### 3.1. Rationale for Performing ANOVA

We conducted ANOVA to evaluate the effectiveness of the predictor variables (F1, F2, F3, and F5) in explaining variation in job stress levels. By examining the statistical significance of the overall model, ANOVA helps determine whether these predictors collectively provide a meaningful explanation of the dependent variable, JSI. This analysis is essential for understanding the degree to which these specific factors impact job stress, guiding further interpretation of individual predictor contributions and the practical implications for managing job stress.

Table 8 provides the regression coefficients for the predictors (F1, F2, F3, and F5) in the model, with Job Stress Index (JSI) as the dependent variable. This regression was conducted through the origin, meaning that no intercept is included in the table.

- F1 has a coefficient of 1.086, meaning that each unit increase in F1 is associated with an increase of 1.086 in JSI.
- F2 has a coefficient of 1.219, suggesting that it contributes more strongly to JSI than other factors.
- F3 and F5 both have a coefficient of 1.147, indicating similar impacts on JSI.
- Also, Table 8 shows that the standardized values allow for comparison across predictors, showing the relative contribution of each predictor to JSI. F1 has the largest standardized beta (0.341), suggesting it has the strongest relative effect on JSI among the four predictors.

### 3.2. Rationale for Conducting the Analysis

We performed this analysis to identify and quantify the specific contributions of each predictor (F1, F2, F3, and F5) to job stress levels. Understanding these coefficients allows us to evaluate which factors strongly influence JSI and the magnitude of their effects, which is critical for targeted interventions in managing job stress.

In our model, an R^2^ value of 0.999 indicates that these predictors account for nearly all the variance in JSI, which suggests a strong model fit. The high F-value and the *p*-value of 0.000 indicate that the model is statistically significant, supporting its validity and appropriateness.

Factor loadings (standardized beta coefficients) represent the relative strength of each predictor’s relationship with the dependent variable, JSI. In this model, we prioritize factor loadings above 0.30 as an indication of meaningful contribution, with F1 having the strongest relative effect (beta = 0.341). This threshold is commonly used in social sciences to gauge the practical significance of each factor in a model.

The statistical significance of each predictor (*p* < 0.001 for all predictors) indicates that each variable contributes meaningfully to explaining job stress levels. This criterion ensures that we only include predictors that have a statistically reliable impact on JSI.

Hence, the high R^2^ and adjusted R^2^, significant F-statistic, and factor loadings all confirm a strong model fit, meeting the criteria for model appropriateness. Each predictor contributes significantly, as demonstrated by the factor loadings above the practical threshold and the statistically significant *p*-values. Together, these indices support the fact that the model provides a robust and interpretable explanation of the factors influencing job stress levels (as captured by the JSI).

Table 9 displays the frequency and percentage of dentists who found the JSI items to cause significant job stress. The occurrences of stressful encounters are presented based on the frequency of times the item received scores of “very stressful = 4” and “most stressful = 5”. “Managing uncooperative or noncompliant patients” was highlighted as the top stressor by 33 (31%) of the dentists, whereas dealing with “interpersonal issues with colleagues” was identified as the least common work-related stressor. Six out of nine patient-related factors were identified as top stressors, while all items related to time pressure were also among the main stressors.

**Figure 2:**
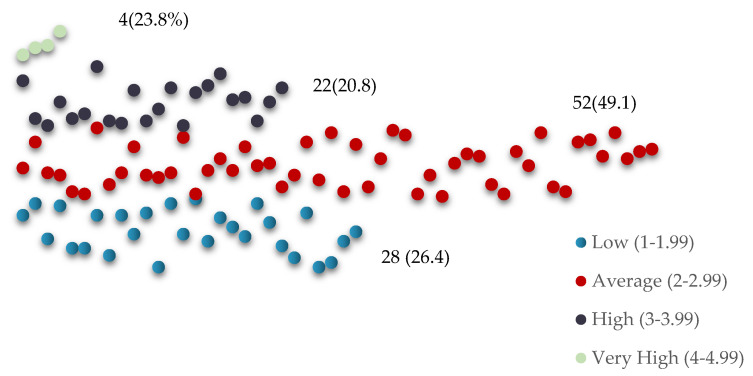
Distribution of PHCC dentists according to job stress levels.

In Figure 2, the dentists were categorized into four groups based on job stress, with 26.4% experiencing low job stress, 49.1% facing an average level of stress, and 20.8% dealing with high levels of stress. A considerable number of dentists, specifically 3.8%, experienced notably high job stress levels.

Results from the study revealed that patient-related factors, job conditions, health system reforms, job characteristics, and time pressure are significant contributors to job stress in the PHCC dental setting. Data analysis demonstrated a strong correlation between job stressors and perceived stress; based on the test results, the null hypothesis can be rejected.

## 4. Discussion

Dentists frequently experience stressful situations that crucially require coping ability to eradicate adverse events during care. The study revealed that 69.8% of the dentists reported experiencing higher levels of perceived stress compared to their counterparts in Iran (50.87%) (Abdi et al., 2022) [25], Iraq (84.3%) (Al-Shaikhli et al., 2022) [26], the UK (54.9%) (Collin et al., 2019) [16], and Mexico (67.8%) (Pozos-Radillo et al., 2014) [27]. These results are consistent with findings from previous studies indicating that dentists frequently experience increased stress in their work environment (Almasri 2020) [19]. A significant correlation between perceived stress (PSS) and sociodemographic variables was observed in different age groups (6.80 > 3.93).

Research consistently indicates that older individuals have lower levels of psychological stress owing to their wealth of experience over the years, enhanced ability to cope with challenging situations, advanced educational achievements like postgraduate certification, and improved financial security (Siddiqui et al., 2022) [28]. In contrast to these results, senior dentists in Saudi Arabia documented increased stress levels (Shaikh et al., 2023) [29]. Younger dentists often report higher stress. They are keen to enhance their skills and self-assurance. They have harbored a fear of making mistakes because they uphold a high standard of care and strive to carry out all tasks flawlessly, resulting in them feeling a little unsure and isolated, especially for those who work outside their home country (Pattranukulkit et al., 2023) [30]. Female dentists repeatedly experience increased levels of stress, a finding that is further supported by this research. The heightened level of stress experienced by females can be traced back to the numerous tasks they typically juggle in both their professional and personal lives, rendering them more susceptible to psychological strain. Studies generally indicate that both male and female dentists face elevated levels of stress, potentially harming their mental health.

Of the dental professionals in this study, PHCC restorative dentists (*n* = 4) displayed elevated perceived stress levels that ranged between 17 and 25, and a mean job stress score of 3.27, though this might indicate a limitation of the study since the sample size of restorative dentists is too small to draw a decisive conclusion, needing deeper investigation. The role ambiguity of restorative dentists may have led to some overlap in job responsibilities with general dentists. Overall, general dentists experienced higher perceived stress (mean = 18.25, SD = 6.35) compared to specialist dentists (mean = 17.68, SD = 5.87), which is a common finding across dental practices, especially for those active in public care (Collin et al., 2019; Pattranukulkit et al., 2023; Pop-Jordanova et al., 2013) [16,30,31].

Heightened stress levels experienced by dentists in leadership positions may stem from the dual demands of clinical practice and managerial responsibilities. Dentists who have recently joined the PHCC demonstrate increased stress as they adapt to their new work environment and navigate interactions with their supervisors, a phenomenon highlighted in the findings of Asif et al. (2022) [32]. Additionally, limited opportunities for career advancement may contribute to their feelings of pressure, as noted in another study (Pattranukulkit et al., 2023) [30].

A comparison between healthy dentists (*n* = 72) and those with medical conditions (*n* = 34) reveals similar chronic stress levels when compared to dentists from Thailand with underlying health issues (Pattranukulkit et al., 2023) [30]. Research indicates that dentists who consistently get more than 8 h of sleep experience lower stress levels. Numerous studies have confirmed the correlation between sleep duration and stress levels among dental professionals (Badrasawi et al., 2024) [33].

The Job Stress Inventory (JSI) previously applied to both public and private Thai dentists was demonstrated to be a reliable tool for evaluating job-induced stress in PHCC dental settings (Cronbach’s alpha ≥ 0.70). This represents a positive development in comprehending and managing workplace stress. Overall, dentists tended to experience an average level of job stress, though 24.5% of the dentists had above-average levels compared to dentists from the UK (39%) (Kemp 2024) [34] and the KSA (83.4%) (Anzar et al., 2023) [35]. In contrast to the findings of Song and Kim (2019) [36], the outcomes of this study did not reveal a significant correlation between sociodemographic factors and job stress predictors.

Findings from previous studies confirm the relationship between job stress and the chronic stress levels experienced by dentists. A significant positive link between chronic stress levels and work-related stress (rs = 0.686, *p* < 0.001) has been shown in Thai dentists. This suggests that the stress experienced in a dentist’s professional life is intricately connected to their overall stress, where work-related factors play a substantial role (Pattranukulkit et al., 2023) [30]. Bhat and Nyathi (2019) [23] utilized the PSS and the WSID scale to present further evidence of this relationship.

This research, on the other hand, provides additional evidence of the correlation between chronic stress felt by dental practitioners and contributing factors from their workplace (r = 0.607, *p* < 0.001). The results decisively support Hosanguan’s (2007) [24] and emphasize the significant impact of patient-related factors and healthcare system changes on work-related stress. The high correlation between patient-related factors and job stress (r = 0.864, *p* < 0.001) highlights the overwhelming stress experienced by dentists as they manage complex patient needs and maintain high clinical and organizational standards while attempting to moderate their demanding workload. A similar correlation was observed among dental practitioners from South Africa (r = 0.62; *p* < 0.01) (Bhat and Nyathi 2019) [23].

The top ten job stressors reported by PHCC dentists mainly relate to patient care and time constraints, underscoring the ongoing significance of patient management as a primary concern for dental professionals (Afsharinia et al., 2023) [37]. Dentists may feel discouraged when faced with challenging or uncooperative patients, particularly those with unrealistic expectations. They strive to deliver high-quality care and meet the needs of their patients, especially when dealing with anxious individuals. The anxiety surrounding the possibility of complaints and legal issues can be overwhelming and distressing for many dentists (Large 2020) [38]. Consequently, studies suggest that conducting screenings for apprehensive and challenging patients could help manage stressful interactions (Goetz et al., 2019) [15].

Time pressure significantly contributes to job stress, particularly in primary care settings. Dentists who deal with a high patient flow often find themselves running behind schedule. This demanding work environment can lead to both physical and psychological issues, ultimately jeopardizing their health and well-being (Kemp 2024) [34]. Adding to this, the long hours at work can make it challenging to spend time with family and friends.

The impact of health system reform on increased job stress levels consolidates the profound influences work culture has on the thoughts and behaviors of workers. Consistent with other research findings, PHCC dentists reported facing challenges in managing additional time spent drilling and documenting patient records. They also have to report to multiple layers of management and adapt to changes within the dental system. Promoting communication and time management skills is crucial for effective patient care and smooth operations within a dental practice. Dentists who work in healthcare corporations experience anxiety stemming from rules and regulations in their organizations (Pattranukulkit et al., 2023), [30] in addition to their fear of patient complaints or misfortunate incidents that increase their concerns about work-related instability. These fears highlight the need for efficient reforms in organizational culture that involve building sustainable teams and creating a supportive and cohesive work environment where staff members contribute to the practice’s success. Promoting professional leadership that harnesses improvement and innovation can lead to better integration between dental practice and employment (Gallagher et al., 2021) [39].

Dental treatment is frequently associated with concerns about cross-infection and discomfort experienced by patients. The undesirable image of being “pain inflictors” and patients’ “dental phobia” contribute to increased anxiety and chronic stress in dental professionals (Henríquez-Tejo and Cartes Velásquez 2016) [40]. As stress levels rise among dentists, there is a significant risk of developing mental health issues, which can adversely affect both their personal and professional lives.

Stress is unavoidable in dental practice, stemming from various work-related factors. Understanding the job factors that contribute to stress is pivotal in preventing its adverse effects on the physical and mental well-being of dental professionals and the subsequent implications on productivity and patient safety. Research emphasizes the need to raise awareness about the potential consequences of stress and to implement effective stress management and tracking programs through organizational intervention (Gallagher et al., 2021) [39] and continuous education, with the goal of enhancing awareness and generating positive attitudes toward coping with stress to maintain a healthy work–life balance (Al-Zubair et al., 2015) [20].

Although this study has limitations, the findings offer important insights into the prolonged psychological stress experienced by PHCC dentists. They highlight how working conditions affect mental well-being. Additionally, the results shed light on the chronic stress levels of dentists in public and primary care settings in Qatar and other countries with similar demographic characteristics in their dental workforce.

## 5. Conclusions

This study aimed to unveil the correlation between perceived stress and contributing job factors. The study demonstrated increased perceived chronic stress levels among a cohort of dentists at PHCC. Approximately two-thirds of the surveyed dentists reported experiencing high (38.7%) and severe (33.1%) levels of chronic stress, mainly related to job factors. Restorative and general dentists reported comparatively higher levels of perceived stress. Older dentists showed a decrease in perceived stress levels. In general, dentists exhibited average levels of job stress. The analysis of the results indicated that patient-related issues, job conditions, health system reforms, and time pressure significantly contribute to elevated stress levels. The frequent sources of stress in PHCC dental practice include coping with uncooperative or noncompliant patients, having excessive workloads, and managing patient flow and documentation. Chronic stress and job-induced stress are prevalent in various dental work settings.

To enhance productivity and well-being, primary care dentists could benefit from the implementation of organizational strategies focused on employee health (Hart, 2002) [41] and a thorough review of job content and patient flow (Akanji 2013) [42]. Enhancing the capabilities of dental staff through incentives and rewards, alongside continuous education and training programs aimed at improving communication and time management skills, could significantly alleviate job-induced stress.

### Limitations

The results cannot be applied to all dentists in Qatar since this study only looked at those employed by Primary Health Care Corporation. Furthermore, the number of participants at each level of stress in certain specializations might not be a reliable indicator of the stress levels in such categories. That data do, however, provide insightful information about the stress levels of public care dentists in the Arab world who share comparable demographics with other dental professionals.

## Figures and Tables

**Table 1:** Demographic information of PHCC dentists.

	N ^1^	%
Specialty		
GP Dentist	75	70.8
Specialist Dentists	31	29.2
Pediatric Dentist	15	14.2
Endodontist	6	5.7
Oral Surgeon	4	3.8
Periodontist	2	1.9
Restorative Dentistry	4	3.8
2. Dental Lead		
Yes	22	20.8
No	84	79.2
3. Sex		
Males	61	57.5
Females	45	42.5
4. Age		
Up to 45 Years	66	62.3
Above 45 Years	40	37.7
45–60 Years	37	34.9
Above 60 Years	3	2.8
5. Marital Status		
Married	99	93.4
Unmarried	7	6.6
Single	4	3.8
Divorced, Separated	3	2.8
6. Time of Work at PHCC		
Up to 5 Years	36	34.0
6–10 Years	27	25.4
11–15 Years	15	14.2
Longer 15 years	28	26.4
7. Engagement in Physical Activity		
Yes	76	71.7
No	30	28.3
8. Hours of Sleep		
<8 h	101	95.3
>8 h	5	4.7
9. General Health		
No Medical Condition	72	67.9
Medically Compromised	34	32.1
Diabetes	4	3.8
Hypertension or Cardiovascular Disease	13	12.3
Asthma	4	3.8
Rheumatoid Arthritis	-	-
Chronic Liver Disease	1	0.9
Other	12	11.3

^1^ *n* = 106.

**Table 2:** Perceived stress vs. sociodemographic variables for PHCC dentists.

	Mean ^1,2^	SD	* *p*-Value
Specialty			0.89
GP Dentist	18.25	6.35	
Specialist Dentists	17.68	5.87	
Pediatric Dentist	17.87	5.08	
Endodontist	15.33	5.75	
Oral Surgeon	18.50	1.29	
Periodontist	17.50	2.12	
Restorative Dentistry	19.75	3.59	
2. Dental Lead			0.62
Yes	18.64	6.30	
No	17.94	5.79	
3. Sex			0.35
Male	17.62	6.12	
Female	18.71	5.52	
4. Age			* 0.01
Up to 45 Years	19.21	5.47	
Above 45 Years	16.23	6.11	
45–60 Years	16.54	6.09	
Above 60 Years	12.33	6.03	
5. General Health			0.97
Married	18.06	5.99	
Unmarried	18.19	5.76	
Single	18.75	4.79	
Divorced, Separated	18.00	3.46	
6. Time of Work at PHCC			0.39
Up to 5 Years	19.28	6.23	
6–10 Years	17.78	5.68	
11–15 Years	18.27	3.88	
Longer 15 years	16.75	6.41	
7. Engagement in Physical Activity			0.52
Yes	17.86	5.95	
No	18.67	5.72	
8. Hours of Sleep			0.33
<8 h	18.21	5.91	
>8 h	15.60	4.83	
9. General Health			0.97
No Medical Condition	18.24	5.23	
Medically Compromised	18.02	5.92	
Diabetes	18.75	3.30	
Hypertension or Cardiovascular Disease	17.69	10.17	
Asthma	16.00	6.98	
Rheumatoid Arthritis	-		
Chronic Liver Disease	20.00	0.00	
Other	17.5	4.76	

* *p* < 0.05. ^1^ *n* = 106. Mean PSS score: the higher the stress score, the more stress. ^2^ R: 0–40.

**Table 3:** ANOVA test of the PSS for age groups.

Source of Variation	SS	df	MS	F	*p*-Value	F Crit
Between Groups	222.231	1	222.231	6.80163	0.01045	3.93244
Within Groups	3398.01	104	32.6731			
Total	3620.24	105				

*p* < 0.05. *n* = 106.

**Table 4:** Factor analysis, mean and standard deviation (SD), and internal consistency (Cronbach’s alpha) of the Job Stress Inventory (JSI) for PHCC dentists.

	Number of Items	Mean ^1,2^	SD	Eigen- Values	% of Variance Explained	Cronbach’s Alpha	** *p*-Value
Factor 1	Patient-Related (9)	2.54	1.16	13.03	43.42	0.89	** 0.002
Factor 2	Job Condition (5)	2.29	1.20	2.14	7.15	0.86	* 0.04
Factor 3	Health System Reform (6)	2.35	1.14	1.69	5.63	0.87	** 0.001
Factor 4	Job Characteristics (4)	2.26	1.15	1.42	4.74	0.73	** 0.005
Factor 5	Time Pressure (6)	2.66	1.22	1.33	4.45	0.91	0.59
Overall	(30)	2.45	1.18	19.62	65.39	0.95	** 0.023

* *p* < 0.05. ** *p* < 0.01. ^1^ Mean value of Job Stress Inventory: the higher the score, the more stress. ^2^ JSI range: 1–5. Note: Cronbach’s alpha significant at α ≥ 0.70.

**Table 5:** Pearson correlation between job stress and perceived stress of PHCC dentists.

PSS	PSS	F1	F2	F3	F4	F5	JSI
	1	0.533 **	0.472 **	0.555 **	0.476 **	0.499 **	0.607 **
F1	0.533 **	1	0.535 **	0.653 **	0.572 **	0.696 **	0.864 **
F2	0.472 **	0.535 **	1	0.623 **	0.635 **	0.659 **	0.800 **
F3	0.555 **	0.653 **	0.623 **	1	0.615 **	0.653 **	0.842 **
F4	0.476 **	0.572 **	0.635 **	0.615 **	1	0.651 **	0.785 **
F5	0.499 **	0.696 **	0.659 **	0.653 **	0.651 **	1	0.883 **
JSI	0.607 **	0.864 **	0.800 **	0.842 **	0.785 **	0.883 **	1

** Correlation is significant at the 0.01 level (2-tailed); range: −1 (negative) to +1 (positive). A value of (0) indicates no correlation.

**Table 6:** Model summary.

Model 1	R	R Square ^b^	Adjusted R Square	Std. Error of the Estimate	R Square Change	F Change	df1	df2	Sig. F Change
	1.000 ^a^	0.999	0.999	2.37458	0.999	27,748.028	4	102	0.000

^a^ Predictors: F5, F2, F3, F1. ^b^ For regression through the origin (the no-intercept model), R Square measures the proportion of the variability in the dependent variable around the origin explained by regression. This cannot be compared to R Square for models that include an intercept.

**Table 7:** ANOVA ^a,b^.

Model 1		Sum of Squares	df	Mean Square	F	Sig.
	Regression	625,843.859	4	156,460.965	6.80163	0.000 ^c^
	Residual	575.141	102	5.639		
	Total	626,419.000 ^d^	106			

^a^ Dependent variable: JSI. ^b^ Linear regression through the origin. ^c^ Predictors: F5, F2, F3, F1. ^d^ This total sum of squares is not corrected for the constant because the constant is zero for regression through the origin.

**Table 8:** Coefficients ^a,b^.

Model 1	Unstandardized Coefficients			Standardized Coefficients		
		B	Std. Error	Beta	t	Sig.
	F1	1.086	0.042	0.341	25.665	0.000
	F2	1.219	0.068	0.197	17.916	0.000
	F3	1.147	0.065	0.224	17.685	0.000
	F5	1.147	0.061	0.255	18.816	0.000

^a^ Dependent variable: JSI. ^b^ Linear regression through the origin.

**Table 9:** Frequency and percentage of PHCC dentists who identified the JSI items as “above average” in terms of stress ^1^.

**Factor 1: Patient-Related (9)**	**Frequency**	**%**	**Rank**
1. Coping with difficult patients	26	25%	6
2. Patients have unrealistic expectations	26	25%	6
3. Treating fearful patients	16	15%	15
4. Patients are dissatisfied with dental care	16	15%	15
5. Coping with uncooperative/noncompliant patients	33	31%	1
6. Fear of making mistakes	27	25%	5
7. Risk of complaint/litigation by patients	28	26%	4
8. Managing children	18	17%	13
9. Patients being late or missing appointments	23	22%	
**Factor 2: Job condition (5)**			
10. Lack of fairness from supervisor	25	24%	7
11. Interpersonal problems with colleagues	11	10%	20
12. Inappropriate physical working conditions	18	17%	4
13. Equipment breakdown and defective materials	14	13%	17
14. Interference of illness with care delivery	16	15%	15
**Factor 3: Health system reform (6)**			
15. Too much paperwork/documentation	30	28%	3
16. Worried about adaptation to changes in dental system	15	14%	16
17. Lack of proper supervision	14	13%	17
18. Lack of time for catch up with new technology	12	11%	19
19. Being supervised by several managements	19	18%	12
20. Having to perform differently from your ideal skills	16	15%	15
**Factor 4: Job characteristics (4)**			
21. Feeling of isolation in practice	12	11%	19
22. Inflicting pain	17	16%	14
23. Repetitive nature of work	13	12%	
24. Risk of cross-infection	21	20%	11
**Factor 5: Time pressure (6)**			
25. Working under constant time pressures	25	24%	7
26. Working behind schedule	22	21%	10
27. Interference of work with private/family life	27	25%	5
28. Too much work/patients	31	29%	2
29. Lack of time for maintaining social relations	24	23%	8
30. Long working hours	27	25%	5

^1^ Above average scores are 4 “very stressful” and 5 “most stressful”.

## Data Availability

The data that support the findings of this study are available from the corresponding author on reasonable request.
